# Ultrarobust Actuator Comprising High-Strength Carbon Fibers and Commercially Available Polycarbonate with Multi-Stimulus Responses and Programmable Deformation

**DOI:** 10.3390/polym16081144

**Published:** 2024-04-19

**Authors:** Jie Sheng, Shengkun Jiang, Tie Geng, Zhengqiang Huang, Jiquan Li, Lin Jiang

**Affiliations:** 1 Henan Provincial Engineering Laboratory of Automotive Composite Materials, School of Mechanical and Electrical Engineering, Henan University of Technology, Zhengzhou 450001, China; 13253355460@163.com (J.S.); jsk18538392013@163.com (S.J.); 2 Henan International Joint Laboratory of Carbon Composition Material, School of Mechanical and Electrical Engineering, Henan University of Technology, Zhengzhou 450001, China; 3 Zhong Yuan Institute, Zhejiang University, Zhengzhou 451191, China; hzq124@gmail.com; 4 College of Mechanical Engineering, Zhejiang University of Technology, Hangzhou 310023, China; lijq@zjut.edu.cn

**Keywords:** actuators, high strength, programmability, carbon fiber, multi-stimuli response

## Abstract

Polymer-based actuators have gained extensive attention owing to their potential applications in aerospace, soft robotics, etc. However, poor mechanical properties, the inability of multi-stimuli response and programmable deformation, and the costly fabrication procedure have significantly hindered their practical application. Herein, these issues are overcome via a simple and scalable one-step molding method. The actuator is fabricated by hot-pressing commercial unidirectional carbon fiber/epoxy prepregs with a commodity PC membrane. Notable CTE differences between the CF and PC layers endow the bilayer actuator with fast and reliable actuation deformation. Benefiting from the high strength of CF, the actuator exhibits excellent mechanical performance. Moreover, the anisotropy of CF endows the actuator with design flexibility. Furthermore, the multifunction of CF makes the actuator capable of responding to thermal, optical, and electrical stimulation simultaneously. Based on the bilayer actuator, we successfully fabricated intelligent devices such as light-driven biomimetic flowers, intelligent grippers, and gesture-simulating apparatuses, which further validate the programmability and multi-stimuli response characteristics of this actuator. Strikingly, the prepared gripper possesses a grasping capacity approximately 31.2 times its own weight. It is thus believed that the concept presented paves the way for building next-generation robust robotics.

## 1. Introduction

Polymer-based actuators, which can convert other forms of energy into mechanical deformation through external physical or chemical stimuli [1,2,3], have gained extensive attention owing to their potential applications in aerospace [4], soft robotics [5,6], artificial muscles [7,8], and so on. The current actuators are mostly fabricated by hydrogels [9,10], elastomers [11,12], shape-memory polymers [13], and liquid crystal [14,15]. A variety of active fillers are introduced to these materials to enhance the actuating performance of actuators, including MXenes [16,17], cellulose microfibrils [18,19], graphene [20], carbon nanotubes [21], and silver nanorods [22]. Despite the great performance improvements of actuators over the past few decades, the high cost of polymeric materials and active fillers, as well as the complex fabrication process, seriously restrict the large-scale use of actuators. The poor thermal–mechanical performance of actuators also significantly hinders their practical application. Moreover, reliable multi-stimuli-responsive programmable actuators also need to be further researched to adapt to complex environments.

Plants offer a perfect solution by introducing rigid, directionally aligned cellulose fibrils into the cell wall [23], thereby inducing anisotropic swelling and shrinking in tissues and realizing on-demand shape changes [24,25]. Inspired by this, aligned nanoparticles [26], graphene [27] and its derivatives, carbon nanotubes [28], and stripes [29] are introduced into the polymeric matrix to produce programmable actuators with complex shape changes. Additionally, molecular alignment in liquid crystal polymer networks could also be used to construct programmable actuators [13]. However, the mechanical strength of these actuators is still limited by the discontinuous character of the aligned fillers and the low strength of the polymeric crosslinking network. Incorporating aligned continuous fibers should be a good way to improve the mechanical properties of actuators and maintain their complex deformation abilities. Based on this idea, stiff vinyl fibers [30], polyimide fibers [31], bamboo [32], and purified wood [33] have been incorporated into polymeric materials to fabricate actuators. However, the introduction of these fibers solely serves the purpose of mechanical reinforcement, lacking multi-stimulus response capability. Therefore, it is still a challenging issue to construct an actuator that integrates high thermo-mechanical performance, cost-effectiveness, large-scale production capacity, multi-stimulus response, and complex deformation ability.

Carbon fiber is an appealing candidate for constructing thermo-mechanical robust actuators with multi-stimuli-responsive abilities because of its outstanding properties, including high mechanical properties, excellent thermal stability and conductivity, and remarkable electrothermal and photothermal conversion capabilities [34,35,36]. Moreover, the anisotropy of carbon fiber enables the facile attainment of complex deformations by the actuator fabricated from it. Qu et al. introduced carbon fiber into a graphene/PI composite to fabricate a thin-film actuator. Carbon fiber endows the actuator with enhanced strength, electric-triggered performance, and intricate deformation [37]. However, a two-step molding process, including pasting and coating, is needed to fabricate the actuator. In addition, the complex deformation ability of the actuator after introducing carbon fibers has not been explored and requires further investigation.

Herein, we present a simple and scalable one-step molding method to fabricate a thermo-mechanical robust actuator, integrating multi-stimuli-responsive and programmable deformation abilities. Specifically, commercial unidirectional carbon fiber epoxy resin prepregs and a commodity polymer polycarbonate (PC) were applied as the passive and active layers of the actuator. The difference in coefficients of thermal expansion (CTEs) between the fiber and the polymeric layer in the actuator results in its deformation through external stimuli. The carbon fiber prepreg layer is bonded to the polymer layer by a widely employed hot-pressing molding technique. Benefiting from the excellent adhesive property of epoxy resin, the two layers are successfully bonded together. The proposed actuator is expected to have three distinct advantages: (1) Commercial carbon fiber prepregs, commodity polymers, and the mature molding method offer the actuator the potential for large-scale utility. (2) The high strength and high modulus of carbon fibers can provide a strong actuating force for the actuator. (3) The anisotropic structure and excellent electrothermal and photothermal conversion capabilities of the carbon fibers greatly enhance the designability of actuators.

Based on the above considerations, a CF/PC bilayer actuator was fabricated by the hot-pressing molding technique using carbon fiber prepregs and a commercial PC film. The microstructure, thermo-mechanical properties, and actuating performance under the stimuli of heat, electricity, and light were characterized for the CF/PC bilayer actuator. Furthermore, the deformation ability of the actuator was assessed by controlling the orientation of the carbon fibers. As a demonstration of the concept, we applied the CF/PC actuator in smart equipment, such as bionic flowers, gesture indicators, and five-finger grippers, to execute tasks.

## 2. Experiment

### 2.1. Materials

PC (polycarbonate) membranes with a thickness of 0.05 mm were obtained from Shenzhen Xinhua Thin Film Technology Co., Ltd. (Shenzhen, China). Four different thicknesses of unidirectional carbon fiber epoxy resin prepregs were utilized to investigate the thickness dependence of the actuating performance for the CF/PC bilayer actuator. The ply thicknesses were 0.06 mm, 0.11 mm, 0.15 mm, and 0.2 mm. The carbon fibers in the prepregs were T700, with a diameter of 7 μm. All the prepregs were purchased from Weihai Guangwei Composites Co., Ltd. (Weihai, China). The alcohol solvent was acquired from Macklin Reagent Company, and the mold-release agent solvent was purchased from Dongguan Mccos Composite Materials Co., Ltd. (Dongguan, China).

### 2.2. Preparation of the CF/PC Bilayer Actuator

The carbon fiber/polycarbonate (CF/PC) bilayer actuators were fabricated by the hot-press molding technique. Firstly, the prepregs were placed on the surface of the lower mold at different angles (0°, 15°, 30°, 45°, 60°, 75°, 90°). Subsequently, the PC membranes were laid upon the membranes. The two layers were adhered together by hot-pressing with a pressure of 4 MPa at 135 °C for 30 min. Corresponding to four thicknesses (0.1 mm, 0.15 mm, 0.2 mm, 0.25 mm), the CF/PC bilayer actuators were labeled as PCF-1, PCF-2, PCF-3, and PCF-4, respectively. Table 1 presents the thickness-related parameters of these CF/PC bilayer actuators.

### 2.3. Characterization

#### 2.3.1. Morphology of the CF/PC Actuator

The microstructure of the CF/PC bilayer actuator was characterized by scanning electron microscopy (SEM) (SU8010, Hitachi, Chiyoda, Tokyo). Before testing, the samples were coated with gold to increase their conductivity. SEM images were obtained at an acceleration voltage of 5 kV.

#### 2.3.2. Dynamic Mechanical Analysis

The dynamic thermo-mechanical properties of the CF/PC bilayer actuators were analyzed by a dynamic mechanical analyzer (DMA, Q800, TA Instruments, New Castle, DE, USA). The samples were tested in tensile mode with a specimen dimension of 30 mm × 5 mm. The loading frequency was fixed at 1 Hz, and the testing temperature was increased from 30 °C to 180 °C at a heating rate of 5 °C/min.

#### 2.3.3. Mechanical Property Characterization

The tensile properties of the CF/PC bilayer actuators were characterized by a universal testing machine (DNS100, GuantengAutomation Technology Co., Ltd., JiLin, China). The dimensions of the test specimens were uniformly set at 250 mm × 25 mm. All tests were conducted at room temperature with a loading rate of 1 mm/min. At least five samples were tested in each group to ensure the accuracy and repeatability of the data.

#### 2.3.4. Thermal Stimulus Response Behavior

To analyze the actuation performance of the CF/PC bilayer actuators under thermal stimulus, a high-precision constant-temperature heating platform, model SET, was utilized for temperature control. Thermal actuation tests were conducted on samples with varying thicknesses and fiber angles, heating from ambient temperature to 80 °C, 100 °C, 120 °C, 140 °C, 160 °C, and 180 °C. Each temperature was held stable before initiating the thermal response operation to minimize temperature discrepancies. The CF/PC bilayer actuators exhibiting anisotropic behavior were prepared by cutting carbon fiber prepreg materials from PCF-1 at various angles (0°, 15°, 30°, 45°, 60°, 75°, 90°).

#### 2.3.5. Light Stimulus Response Behavior

Light stimulus response behavior was performed using a commercial Philips heat lamp (100–375 W, wavelength: 570–600 nm, Philips, Amsterdam, Netherlands). The actuation performance was conducted under varying light intensities ranging from 0.1 W/cm^2^ to 1.2 W/cm^2^. The dimensions of the test samples were 40 mm × 25 mm × 0.1 mm. Infrared thermal imaging tests were carried out using a Testo 865 infrared camera (Testo, Lenzkirch, Germany) to monitor the temperature change of the samples.

#### 2.3.6. Electric Stimulus Response Behavior

A variable direct current (DC) stabilized power supply (MaiSheng, 15 V, 20 A, Maisheng Power Technology Co., Ltd, Dongguan, China) was employed for electric-stimulus actuation performance testing. The dimensions of the samples were 75 mm × 25 mm × 0.15 mm. The actuation tests were conducted using voltages ranging from 0.15 V to 1.2 V. To induce deformation through electrical heating of the samples, two wires were used to connect the positive and negative terminals of the variable DC stabilized power supply. The magnitude of the current was regulated by controlling the voltage across the carbon fibers.

#### 2.3.7. Bending Curvature

**Figure 1:**
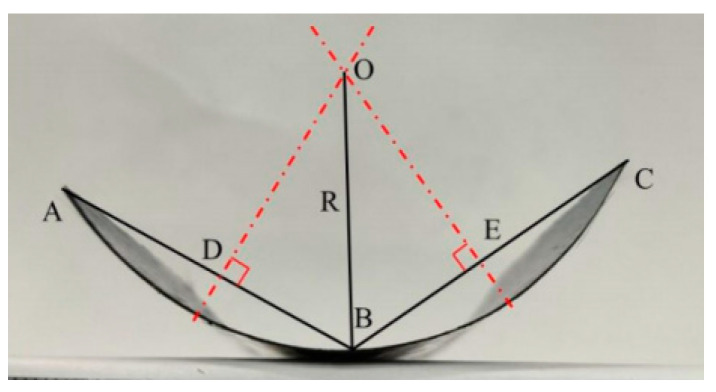
Schematic diagram of curvature radius calculation.

Bending curvature was used to analyze the deformation extent of the CF/PC bilayer actuators. The calculation method for the curvature is shown in Figure 1. Points A and C are located at the endpoints of the bending sample’s arc, while point B is the midpoint of this arc. Points D and E are the midpoints of line segments AB and BC, respectively. Perpendicular bisectors of line segments AB and BC intersect at point O, which is considered the center of the arc. The bending curvature is calculated as 1/R.

#### 2.3.8. Actuation Deformation Rate

**Figure 2:**
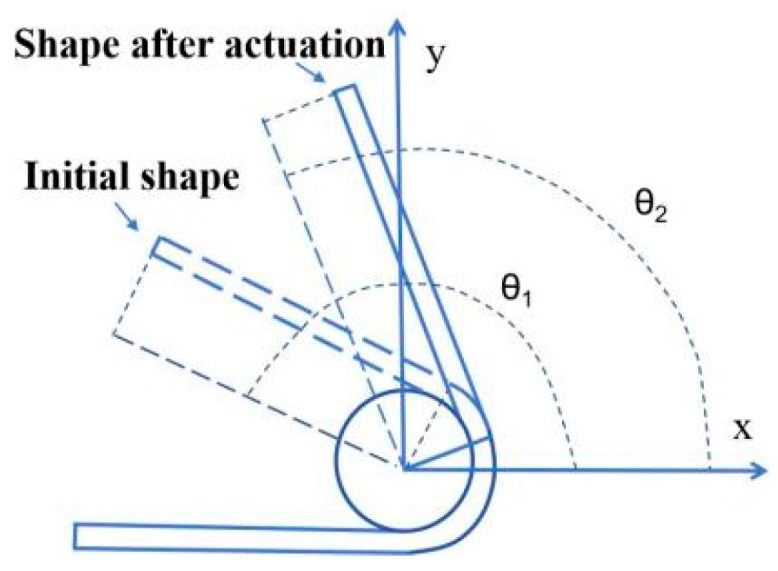
Schematic diagram of actuation deformation rate calculation.

The actuation deformation rate is utilized to quantitatively analyze the shape-changing extent of the actuators. As shown in Figure 2, the actuation deformation rate is calculated using the equation (θ_1_ − θ_2_)/θ_1_, which is the ratio of shape variation during actuation to initial shape.

## 3. Results and Discussion

### 3.1. Fabrication and Characterization

**Figure 3:**
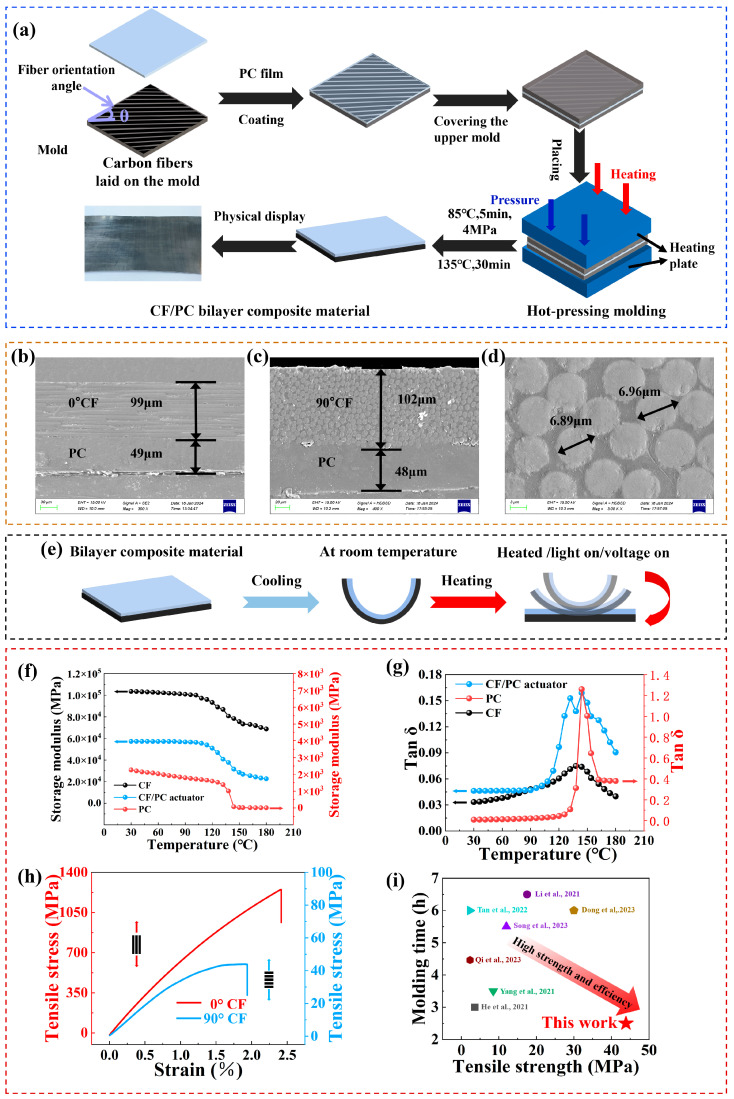
Fabrication and characterization of CF/PC bilayer actuators. (**a**) Schematic illustration of the fabrication process of CF/PC bilayer actuators. (**b**) SEM image of the cross-section of CF/PC actuator in the 0° CF direction. (**c**) SEM image of the cross-section of CF/PC actuator in the 90° CF direction. (**d**) Close-up view of the carbon fiber area. (**e**) Schematical illustration of the actuation principle of CF/PC bilayer actuators. (**f**) Storage modulus of PC film, CF prepreg, and CF/PC bilayer actuator from 30 °C to 180 °C. (**g**) Loss factor of PC film, CF prepreg, and CF/PC bilayer actuator from 30 °C to 180 °C. (**h**) Stress–strain curve of the actuator. (**i**) Comparison of molding time and tensile strength between CF/PC bilayer actuators and other types of actuators [13,18,44,45,46,47,48].

Composites with poor impregnation effects or even with a layered structure are a drawback in the field of traditional composites [38]. We took advantage of this “flaw” and realized the control of the deformation behavior of composites with external stimuli, including thermal, light, and electric. As illustrated in Figure 3a, the CF/PC bilayer actuator was fabricated by laminating carbon fiber prepregs with a PC membrane, followed by a hot-press molding process to create a composite. The details of the preparation are documented in the Experiment section. The fabrication route is stable and suitable for the mass production of smart composite actuators. From the cross-sectional views of the composite plate material in the 0° and 90° fiber directions (Figure 3b–d), it is evident that the structure exhibits a typical bilayer configuration. By enlarging the interface area, the PC resin penetrates into the CF layer and firmly sticks to the CF, thus achieving a stable structure.

This bilayer structure is expected to endow the actuator with rapid response, superior mechanical properties, significant deformation, multi-stimuli responsiveness, and complex shape changes. As shown in Figure 3e, the CF prepreg is selected as the passive layer due to its excellent photothermal and electrothermal conversion efficiency and lower thermal expansion coefficient (0.3 × 10^−6^ °C^−1^) [39]. Conversely, PC is selected for the active layer because of its higher thermal expansion coefficient (5.8 × 10^−5^ °C^−1^) [40]. Upon temperature change, the PC layer exhibits significant volume changes (expansion or contraction), while the volume change of the CF layer is relatively small. Meanwhile, the presence of the CFs limits the expansion or contraction of the surrounding matrix, leading to a stress mismatch between the two layers and causing bending deformation [37]. Additionally, the CF prepreg material not only has lightweight and high-strength characteristics but also provides design flexibility and fatigue resistance, thus ensuring the programmable actuation behavior of the high-strength actuator.

Figure 3f,g present the DMA curves for the PC, the CF/PC actuator, and the carbon fiber prepregs. The storage moduli of the PC and the cured carbon fiber prepregs are significantly different. The CF prepregs exhibit an extremely high modulus of 103 GPa, while the PC has a relatively low modulus of only 2.5 GPa. Due to the reduced contribution of the polymer to the modulus, the maximum elastic modulus of the CF/PC actuator is only 57.1 GPa [41]. For all the samples, the storage modulus gradually decreases as the temperature increases, showing a typical temperature-dependent characteristic. The loss factor is used to describe the transition temperature (T_g_) of the samples. The peaks on the PC and CF curves correspond to their T_g_ values, which are 144 °C and 138 °C, respectively [42]. The CF/PC actuator exhibits two significant peaks, the first corresponding to the T_g_ of the epoxy resin, which is about 133 °C [43]. The second peak corresponds to the T_g_ of the PC matrix, which is about 144 °C.

X-ray photoelectron spectroscopy (XPS) was employed to investigate the composition of the CF/PC bilayer actuators on both sides. As depicted in Figure S1, prominent peaks at approximately 284.3 eV for C 1s and approximately 532.4 eV for O 1s are present on the PC side, while the N 1s peak appeared on the other side, indicating the existence of cured epoxy resin on the CF side. The difference in XPS data between the two sides clearly reveals that the CF/PC actuator has a distinct bilayer structure. Additionally, the thermal stability of the actuator was thoroughly investigated to ensure its suitability in high-temperature environments. Figure S2 illustrates the thermal stability curves and degradation temperatures of the PC, the carbon fiber prepregs, and the CF/PC actuator at approximately 473 °C, 390 °C, and 383 °C, respectively. Due to the influence of epoxy resin in the carbon fiber prepregs, the degradation temperature of the CF/PC actuator is slightly lower than that of the PC film. Generally, the CF/PC bilayer actuator demonstrates excellent thermal stability, remaining quite stable over the actuating test temperature range of 200 °C.

For the bilayer actuator, the mechanical performance is significantly improved by the CF, as demonstrated by the tensile test at room temperature. As shown in Figure 3h, the stress–strain curves with 0° and 90° fiber orientations are compared. When θ = 90°, the sample exhibited a strain of up to 1.93% at a stress level of 43 MPa. Conversely, at θ = 0°, the composite material displayed a strain of up to 2.4% at a significantly higher stress level of 1230 MPa. As depicted in Figure 3i, the CF/PC actuator exhibits significant advantages over most current actuators in terms of molding time and mechanical performance [13,18,44,45,46,47,48]. Specifically, it considerably reduces the molding cycle and enhances production efficiency. Moreover, its mechanical performance surpasses that of conventional actuators due to advancements in carbon fiber composite molding technology and the inherent high-strength characteristics of carbon fiber itself. Notably, compared with various fiber-reinforced actuators currently available, the CF/PC actuator shows excellent performance in stimulus response diversity, response time, and deformation complexity. Consequently, the proposed actuator fabrication route not only ensures its reliable and stable operation under high-load conditions and extreme operating environments but also facilitates its large-scale use.

### 3.2. Thermo-Responsive Actuation Behavior of the CF/PC Actuator

**Figure 4:**
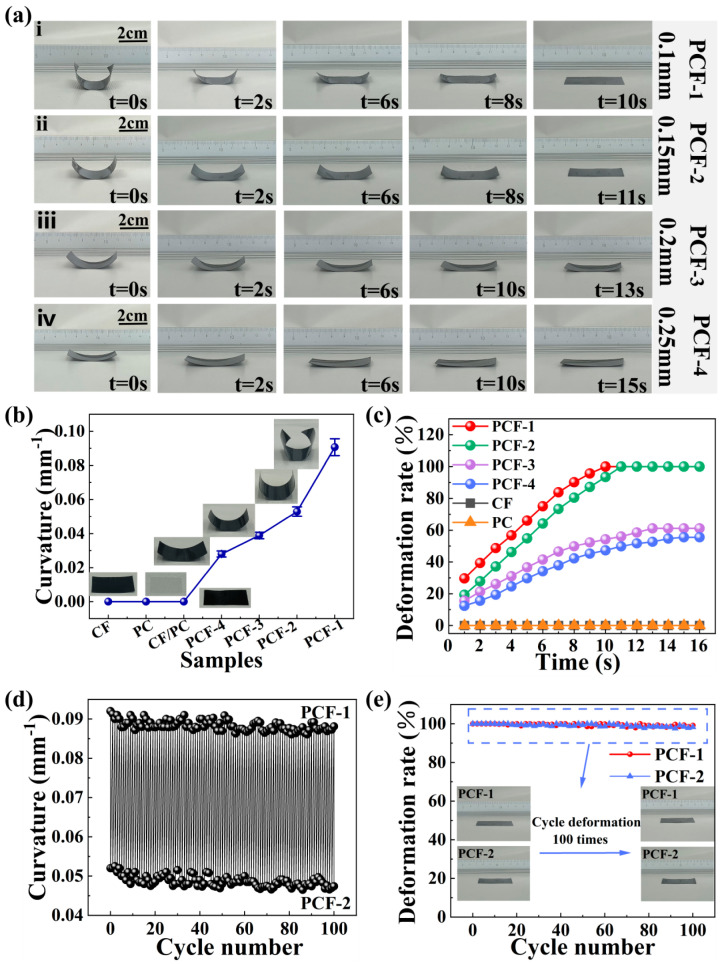
Thermo-responsive actuation behavior of the CF/PC actuators. (**a**) Photographs depicting the deformation of CF/PC bilayer actuators with four different thicknesses at 140 °C (**i**) PCF-1, (**ii**) PCF-2, (**iii**) PCF-3, (**iv**) PCF-4. (**b**) Curvature of various samples at ambient temperature. (**c**) Changes in deformation rate of different samples at 140 °C. (**d**) Variations in curvature of PCF-1 and PCF-2 actuators over 100 cycles. (**e**) Changes in deformation rate of PCF-1 and PCF-2 actuators over 100 cycles.

To achieve a CF/PC bilayer actuator with optimal actuating performance, we fabricated four types of actuators with varying thickness ratios. The influence of thickness ratio on actuator performance was investigated. Real-time thermal deformation photographs of bilayer actuators with different thickness ratios at 140 °C were captured, as shown in Figure 4a(i–iv) and Movie S1. The results show that, when subjected to thermal stimulation, the bilayer structures with different thickness ratios rapidly exhibit significant thermal deformation behavior in a short time. This indicates that as the thickness of the CF layer increased, the deformation extent of the bilayer actuators progressively diminished. To analyze the deformation of the bilayer actuators in greater detail, curvature parameters were employed for quantitative assessment. As illustrated in Figure 4b, in comparison to CF/PC actuators with asymmetric bilayer structures, pure polycarbonate films, cured CF prepreg materials, and structurally uniform CF/PC composites have no actuation deformation under thermal stimulation. This absence of actuation behavior is attributed to the fact that a single matrix layer, a single fiber layer, or uniformly structured bilayer composites do not form the required asymmetric bilayer structures, thus lacking the fundamental actuation capability.

Moreover, as shown in Figure 4c, it was observed that as the thickness ratio of the bilayer actuator decreased, the curvature increased gradually. The curvature for PCF-4 was 0.028 mm^−1^, while the bilayer actuator exhibited the highest curvature (0.092 mm^−1^) when the thickness of the CF layer was reduced to 0.1 mm (PCF-1). The phenomenon can be ascribed to the fact that the increased relative thickness of the CF layer in thicker bilayer actuators enhances the stiffness of the actuators. The increased stiffness implies greater resistance to deformation, thus limiting the flexible bending ability of the actuator [49]. Therefore, the degree of deformation increases as the relative thickness of the CF layer in the actuator decreases. 

In order to further investigate the thermal response of the actuators with varying thickness ratios, the real-time deformation rate at 140 °C was captured to quantify and analyze the differences in the deformation of the actuators. As shown in Figure 4d, neither the cured CF prepreg material nor the PC matrix exhibited any deformation, resulting in a deformation rate of 0. The actuators designed with an asymmetric structure demonstrated significant changes in their deformation rates when exposed to thermal stimuli. It was observed that as the relative thickness ratio increased, the maximum achievable deformation rate decreased. When evaluated based on response speed and deformation rate, the bilayer actuator with a thickness of 0.25 mm (PCF-4) reached only about 50% deformation rate within 16 s, whereas the 0.15 mm thick (PCF-2) bilayer actuator could fully unfold within 11 s, achieving a deformation rate of 100%. This implies that as the relative thickness ratio of the CF layer increases in the CF/PC bilayer actuator, the deformation stress stored in the drive is not enough to promote its actuation.

Furthermore, to assess the deformation stability of the actuators, PCF-1 and PCF-2 samples were subjected to 100 cycles of actuation testing. Figure 4e records the bending curvature range of the PCF-2 bilayer actuator throughout the 100 deformation cycles, varying from 0.0475 mm^−1^ to 0.052 mm^−1^, with a fluctuation amplitude of only 7.57%. The bending curvature range of the PCF-1 bilayer actuator varied from 0.086 mm^−1^ to 0.092 mm^−1^, with a fluctuation amplitude of just 6.52%. This demonstrates that the curvature of the actuators did not significantly deteriorate after repeated deformation. Figure 4e illustrates that after 100 actuation cycles stimulated by thermal triggers, the CF/PC bilayer actuators showed virtually no change in deformation rate, continuing to exhibit outstanding actuation deformation performance. In summary, these findings indicate that the actuator possesses excellent cyclic repeatability and long-term usage stability.

**Figure 5:**
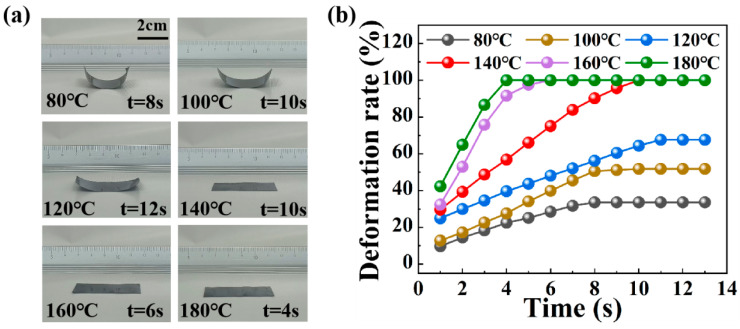
Actuation performance of actuators at different temperatures. (**a**) Photographs of the PCF-1 actuator depicting deformation at various temperatures. (**b**) Changes in the deformation rate of the PCF-1 actuator at different temperatures.

To investigate the impact of temperature on actuation behavior, a broad temperature range of 80–180 °C was utilized to stimulate the actuation deformation of the PCF-1 bilayer actuator. The responsive times were recorded accordingly. As depicted in Figure 5a, with the increase in thermal stimulation temperature, the degree of the actuator reaching the final stable expansion state increases gradually. Notably, at a thermal stimulation temperature of 140 °C, the PCF-1 bilayer actuator only required 10 s to fully unfold. Figure 5b explores the deformation rate over time at varying temperatures within the 80–180 °C range. The deformation rate exhibits an upward trend with the rise in temperature. Specifically, at 120 °C, the PCF-1 bilayer actuator achieved a stable deformation rate of 67.6% within 11 s, and at 140 °C, it reached a 100% deformation rate within 10 s. Remarkably, at 180 °C, the actuator attained a 100% deformation rate in just 4 s. This phenomenon indicates that the deformation of the CF/PC bilayer actuator can fully unfold in high-temperature environments. Furthermore, as the temperature of thermal stimulation increased, the deformation speed of the actuator gradually accelerated. This is primarily attributed to the increase in thermal stress in the high-temperature environment, which facilitates the accelerated deformation rate of the bilayer actuator.

### 3.3. Programmable Actuation Behavior of the CF/PC Actuator

**Figure 6:**
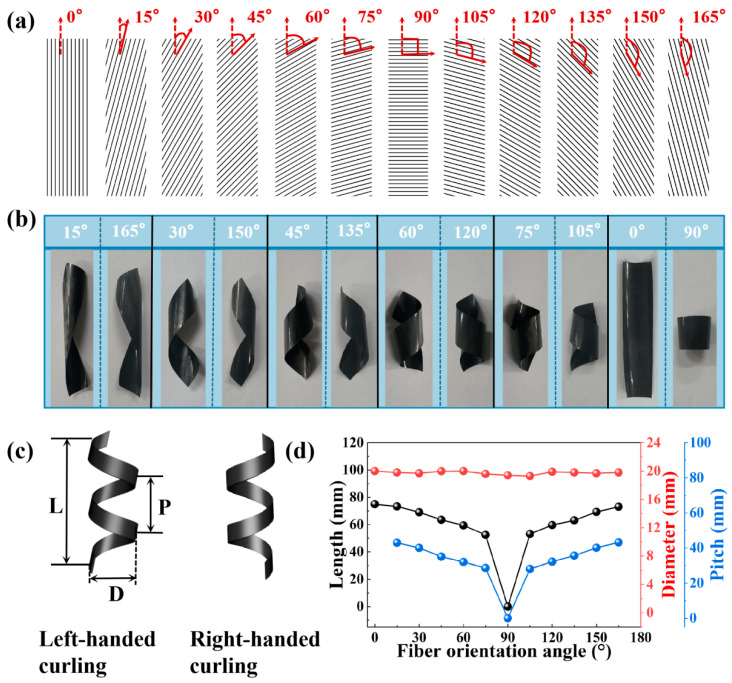
Anisotropic deformation behavior of CF/PC bilayer actuators with varied orientations. (**a**) Schematic representation of rectangular actuators with different fiber orientation directions. (**b**) Structures of rectangular actuators with various fiber directions under thermal stimulation at 140 °C. (**c**) Geometric models of left-handed and right-handed helical curlings. (**d**) Deformation parameters of CF/PC actuators as a function of orientation angle.

Simple actuators are typically confined to basic bending deformations. However, in our CF/PC actuators, stiff carbon fibers exhibit a higher modulus compared to the PC matrix, which can induce the anisotropic swelling or shrinking of the surrounding soft PC matrix. Complex deformation is expected to be achieved by adjusting the directional orientation of the reinforcing CFs. As illustrated in Figure 6a,b, we successfully developed a bilayer actuator with programmable deformation ability by changing the cutting angle (θ) of the anisotropic carbon fiber. The cutting angle is defined as the angle between the longitudinal axis of the rectangular actuator and the alignment direction of the carbon fiber bundles. At various cutting angles (15°, 30°, 45°, 60°, 75°, 105°, 120°, 135°, 150°, 165°), the actuators are capable of curling into spiral structures, forming arch structures along the short axis, or presenting hollow cylindrical structures along the long axis of the rectangular bilayer actuator. Despite the diverse appearances of these programmable deformation modes, they exhibit a consistent deformation direction. Under thermal stimulation, bending and spiral deformations invariably occur perpendicular to the fiber alignment direction of the bilayer actuator. This is attributed to the anisotropy of the CF, which limits deformation along the fiber direction within the surrounding resin matrix, allowing isotropic deformation of the resin matrix in directions perpendicular to the reinforced fiber axis [23]. In addition, either a left-handed or right-handed spiral shape could be obtained by changing the cutting angle. As presented in Figure 6b, the CF/PC bilayer actuator exhibits a left-handed spiral when the cutting angle ranges from 0°to 90°. Conversely, when the cutting angle ranges from 90° to 180°, the actuator forms a right-handed spiral. This discovery highlights the potential of CF/PC bilayer actuators for complex shape-changing control and marks a significant innovation in actuator design.

To quantitatively analyze the impact of fiber orientation angles on the deformation of CF/PC bilayer actuators, we define the relevant parameters as shown in Figure 6c. Here, ‘L’ denotes the axial length, ‘D’ represents the coil diameter, and ‘P’ indicates the pitch. As illustrated in Figure 6d, under the thermal stimulation condition of 140 °C, as the orientation angle increases, the curl degree of the CF/PC bilayer actuator exhibits an ascending trend, while the axial length and the pitch show a gradual descent. For instance, at a cutting angle of 15°, both the axial length and the pitch are relatively large, measuring 73.4 mm and 43.2 mm, respectively. This is because at a fiber angle of 15°, the deformation closely approximates a change along the 0° direction, leading to a more linear deformation and, thus, smaller changes in the fiber length direction. When the fiber orientation angle is increased to 30°, the spiral deformation becomes more pronounced. Further intensification of the curling is observed when the cutting angles reach 45° and 60°. It is important to note that at a cutting angle of 75°, both the axial length and the pitch reach their minimum dimensions, measuring 52.2 mm and 28.6 mm, respectively. This is because as the fiber orientation angle increases, the actuator gradually transforms from linear deformation along the 0° fiber direction to cylindrical deformation perpendicular to the 0° fiber direction, achieving the maximum degree of curl. With the further increase in cutting angle, the actuator begins to transform from a left-handed spiral to a right-handed spiral. Since left-handed and right-handed spiral actuators generate torsional forces of equal magnitude but in opposite directions, their axial lengths and pitches are almost identical [50]. In summary, these results reveal that the diverse structural variations in CF/PC bilayer actuators can be achieved by adjusting the angle of the CF filaments.

### 3.4. Light-Driven Behavior of the CF/PC Actuator

**Figure 7:**
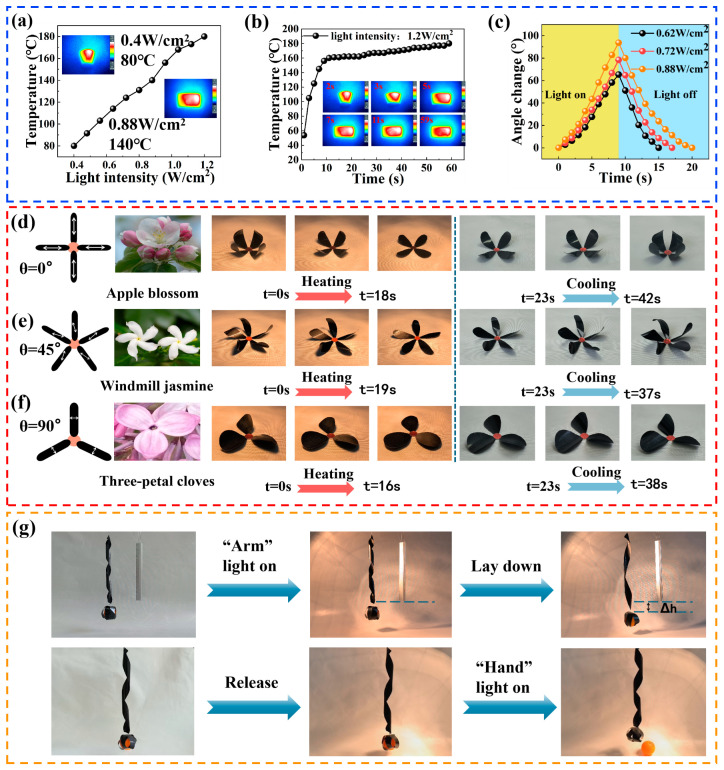
Light-driven behavior of the CF/PC actuators. (**a**) Temperature evolution of the CF/PC bilayer actuator under varying light intensities. (**b**) Real-time temperature and thermal distribution of the actuator under 1.2 W/cm^2^ light irradiation. (**c**) Temperature changes under different light intensities when the light is turned on for 9 s. (**d**–**f**) Deformation of biomimetic flower actuators with multidirectional orientations. (**g**) Intelligent gripper with different deformation structures.

The excellent photothermal effect of the CF is anticipated to confer the CF/PC actuator with light-responsive deformation capability. To investigate the photothermal capacity of the actuator under different light intensities, we placed the PCF-1 actuator in a light intensity range of 0.4 W/cm^2^ to 1.2 W/cm^2^ and recorded the surface temperature changes of the actuator. As shown in Figure 7a, the temperature of the CF/PC bilayer actuator gradually increased with the increase in light intensity. Infrared thermal imaging revealed, that at a light intensity of 0.4 W/cm^2^, the actuator temperature only reached about 80 °C. When the light intensity was increased to 0.88 W/cm², the equilibrium temperature of the actuator reached 140 °C, at which point the deformation of the bilayer actuator could fully recover. These results not only verify that the temperature control of the bilayer actuator can be achieved by adjusting the light intensity, but also confirm that the actuator has excellent responsiveness to light stimulation. Figure 7b records the real-time temperature at a light intensity of 1.2 W/cm^2^. The infrared thermal imaging results demonstrate that the actuator rapidly reaches a temperature of 83 °C within 2 s under continuous illumination. At this stage, the actuator exhibits a curling shape due to its low temperature. As time goes by, the actuator gradually expands. When the illumination lasts for 7 s, the actuator reaches a high temperature of 140 °C and completely expands. Furthermore, the CF/PC actuator stabilizes at an equilibrium temperature of 180 °C within 59 s, indicating its deformation ability at a wide range of temperatures. In summary, these findings not only reveal the significant effect of light intensity on the actuator deformation behavior but also demonstrate the excellent photothermal effect and rapid light-response capability of the actuator.

To further investigate the reversible deformation behavior of the bilayer actuator under light stimulation, the actuator angle change over time was measured under three light intensities of 0.64 W/cm^2^, 0.72 W/cm^2^, and 0.88 W/cm^2^ (shown in Figure 7c). Within 9 s, the maximum angle change of the PCF-1 actuator increased from 78.4° to 93.7° as the light intensity increased from 0.64 W/cm^2^ to 0.88 W/cm^2^. Notably, even under a lower-light-intensity condition (i.e., 0.64 W/cm^2^), a significant angle change of 65.3° was achieved at the same time. When the irradiation was stopped, the PCF-1 bilayer actuator gradually returned to the pre-set bent shape. This observation indicates that the actuator has outstanding programmable deformation ability and has potential applications in controllable bending.

Inspired by the morphological changes of natural flowers under the stimulation of the surrounding environment, three typical bionic flowers were designed in this paper by using the anisotropy of CF. The flower structures were achieved by individually cutting out multiple petals with specific orientation angles on a CF/PC bilayer actuator and precisely gluing them together at specific angles. Figure 7d–f and Movie S2 showcase the patterns of these flowers. When the angle between the CF and the petal axis is 0°, the apple-flower actuator shows a rapid opening process due to the photothermal effect under continuous visible light. All the petals completely open within 18 s. As the visible light is removed and the temperature is reduced, the petals begin to bend inward and curl into a bud-like apple flower within 19 s. When the angle between the CF and the petal axis is 45°, the actuator shows a curled shape similar to a windmill jasmine. Under continuous visible light radiation, the adjacent two petals rapidly expand in a clockwise direction. When the radiation is stopped, they will curl and contract counterclockwise around the pistil. When the angle between the CF and the petal axis is 90°, the actuator shows a curled three-petal lilac structure. During the process of illumination or removal of illumination, the petals show morphological changes of outward expansion and inward contraction. The design cleverly takes advantage of the anisotropy of the fiber material and realizes the conversion of the actuation mode by reasonably assembling the bionic petals, showing excellent light-response characteristics.

To validate the feasibility of CF/PC bilayer actuators in the realm of active coordinated deformation structures, we prepared a combined intelligent gripper with two innovative components. One serves as an arm with dimensions of 260 mm × 20 mm × 0.1 mm. The fibers in the smart arm are oriented at a 60° angle, imparting it with a spiral deformation characteristic. The other one serves as a clamp with dimensions of 120 mm × 18 mm × 0.1 mm. It consists of three rectangular splines intersecting at 60° with an internal fiber angle of 0°. Under visible light, the arm exhibits directional deformation to transport objects, while the gripper contracts to grasp or release objects. As shown in Figure 7g and Movie S3, the upper smart arm extends or contracts the lower clamp under visible light stimuli, successfully realizing the vertical movement of objects. Meanwhile, the lower clamp can grab or release the ping-pong ball under visible light stimuli. It is noteworthy that the gripper itself has a weight of 0.425 g, while the ping-pong ball has a diameter of 39.6 mm and weighs 2.78 g. This indicates that the light-driven intelligent gripper can successfully grab an object approximately six times heavier than itself. Overall, by adjusting the orientation angle and harnessing the photothermal effect of CF in the actuator, we have successfully achieved cooperative coupling between the “arm” and the “hand”, demonstrating a high level of integration in our robotic system.

### 3.5. Electrically Driven Behavior of the CF/PC Actuator

**Figure 8:**
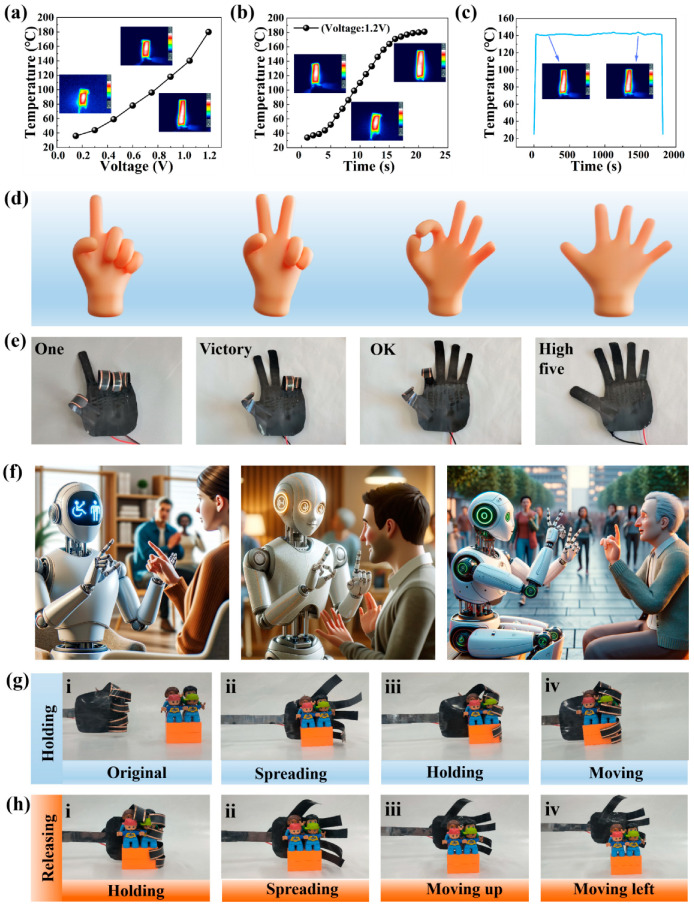
Electrically driven behavior of the CF/PC actuator. (**a**) Temperature evolution of the CF/PC bilayer actuator with increasing voltage. (**b**) Real-time temperature and thermal distribution of the actuator at a voltage of 1.2 V. (**c**) Long-term temperature fluctuations observed in the actuator under a constant voltage of 1.05 V. (**d**,**e**) Schematic diagrams and actual images illustrating gesture models based on electroresponsive behavior of the actuator. (**f**) Application scheme of CF/PC bilayer actuators in communication with individuals who are deaf and communicate using sign language. (**g**,**h**) Programmable movements demonstrated by the five-finger gripper.

To investigate the electrostimulation response behavior of actuators, a series of conductivity tests were conducted to expand their broad applications. Taking the PCF-2 bilayer actuator as a representative, Figure 8a displays its temperature changes under different applied voltages. The temperature of the CF/PC bilayer actuator exhibits an approximately linear increase as the applied voltage rises. Specifically, when the applied voltage is increased from 0.15 V to 1.2 V, the temperature of the PCF-2 actuator rapidly escalates from 36 °C to 180 °C. Based on the Joule heating principle, expressed by the formula Q = U²t/R, with increasing input voltage, the actuator converts more electrical energy into thermal energy, thereby causing the driving deformation behavior of the CF/PC bilayer actuator. This fact not only confirms the congruence between the theoretical principles and the practical experiments but also indicates that the deformation of the actuator can be programmatically controlled. As shown in Figure 8b, the surface temperature of the CF/PC bilayer actuator, as detected by infrared thermal imaging, gradually increases over time under the influence of voltage, leading to progressively greater deformation and ultimately reaching a stable temperature value. Taking the application of 1.2 V to the actuator as an example, the PCF-2 bilayer actuator responds swiftly, rising from ambient temperature to 140 °C within 13 s and reaching a stable temperature of 180 °C in 20 s. This excellent electrothermal conversion performance, achieved at a relatively low input voltage (180 °C at 1.2 V), can be attributed to the low electrical resistance of CFs. This characteristic effectively facilitates the flow of electric current, thus obtaining Joule heating-driven actuation and deformation control. In practical applications, the reliability and stability of an electrothermal actuator are of utmost importance. We assessed the long-term heating stability of the CF/PC bilayer actuator by monitoring prolonged temperature changes at a constant voltage of 1.05 V (as illustrated in Figure 8c). Notably, even after applying external voltage for over 1800 s, the temperature of the actuator remains virtually unchanged, confirming its exceptional stability under extended heating conditions. Therefore, the CF/PC bilayer actuator not only exhibits remarkable electrothermal effects but also enables precise deformation control through voltage adjustment while providing stable heating performance during actuation. This offers valuable insights for relevant application domains.

Given the potential application of actuators in the field of robotics, along with their design flexibility and exceptional actuation performance, we embarked on the design and fabrication of biomimetic actuators to explore their potential, as demonstrated in Figure 8d. Initially, we utilized electroresponse to simulate various hand gestures. Here, we achieved movement variation between different fingers by controlling the electrothermal effect. Specifically, as illustrated in Figure 8e and Movie S4, for gestures like the ‘OK’ sign, we connected two adjacent copper foils on the index and middle fingers. These foils were then connected to another set of copper foils at the ends of the index and middle fingers using positive and negative electrode wires. Subsequently, upon activating the power supply and applying voltage, the temperature of both fingers rapidly increased due to the electrothermal effect, causing them to unfold. Following a similar approach, we successfully achieved several other gestures, such as ‘One’, ‘Victory’, ‘OK’, and ‘High Five’. Based on these characteristics, a series of complex sign language gestures could be constructed to facilitate interaction with individuals who are deaf or hard of hearing (Figure 8f). The accuracy and sophistication in the usage of smart hand gestures not only aid in conveying complex academic concepts but also significantly enhance the learning experience for these students.

Subsequently, to further investigate the functionality of CF/PC actuators, we fabricated a five-finger gripper resembling the size of an adult left hand. As depicted in Figure 8g and Movie S5, due to the excellent electrothermal conversion ability of the actuators, the temperature of the five fingers gradually increased until they were able to fully open, displaying a high-five gesture. Upon the removal of the applied voltage, the fingers swiftly bent and closed together, enabling the gripper to grasp and move objects. The specific process consisted of four stages: (i) the five-finger gripper was maintained at ambient temperature; (ii) the power supply was activated and a predetermined voltage was applied, resulting in the gripper heating up and extending towards an object; (iii) the power supply was subsequently turned off, leading to a gradual decrease in the gripper temperature and its bending motion for grasping the object; (iv) the gripper successfully grasped the object and executed a lateral movement. The gripper in the initial state, as depicted in Figure 8h, securely held an object. After undergoing electrothermal conversion, the fingers of the gripper heated up and unfolded, allowing it to move upwards first and then to the left, resulting in successful object release. It is noteworthy that the electro-actuated five-finger gripper itself weighs a mere 3.395 g, yet it is capable of effectively grasping objects weighing up to 105.8 g. The aforementioned statement implies that the grippers possess a grasping capacity approximately 31.2 times their own weight. The significant finding presented herein demonstrates the substantial potential of the gripper in efficiently handling heavy objects, thereby highlighting its applicability in various robotic applications.

## 4. Conclusions

In conclusion, a novel CF/PC bilayer actuator has been fabricated using a simple and scalable hot-pressing molding technique. The proposed actuator not only demonstrates remarkable mechanical properties but also exhibits high production efficiency, making it highly suitable for large-scale manufacturing. As the relative thickness ratio of the CF layer decreases, the CF/PC bilayer actuator shows a gradually increasing curvature and deformation rate. Even after 100 actuation cycles, the actuator still maintains outstanding actuation deformation performance. Furthermore, programmable and complex shape changes could be achieved by adjusting the angle of the CF filaments in the actuator. Due to the multifunctional properties of CFs, the actuator simultaneously has thermo-, light-, and electroresponsive actuation performance. Utilizing the freedom in shape cutting and specific programmable structures of the CF/PC bilayer actuator, we fabricated a series of biomimetic devices, including light-driven biomimetic flowers, intelligent grippers, and gesture-simulating apparatuses. Moreover, to expand the applicability of the actuator, we designed and produced a five-finger gripper, realizing multifunctional movements under electro-driven actuation. We firmly believe that this CF/PC bilayer actuator, with its superior actuation performance, holds vast potential for applications in aerospace, soft robotics, and biomedical engineering.

## Figures and Tables

**Table 1:** Thickness-related parameters of the CF/PC bilayer actuators.

Name	CF Thickness (mm)	PC Thickness (mm)	Actuator Thickness (mm)
PCF-1	0.05 ± 0.01	0.05 ± 0.002	0.1 ± 0.01
PCF-2	0.1 ± 0.01	0.05 ± 0.002	0.15 ± 0.01
PCF-3	0.15 ± 0.005	0.05 ± 0.002	0.2 ± 0.01
PCF-4	0.2 ± 0.005	0.05 ± 0.002	0.25 ± 0.01

## Data Availability

Data are contained within the article.

## Supplementary Materials

The following supporting information can be downloaded at: https://www.mdpi.com/article/10.3390/polym16081144/s1, Figure S1: XPS curves of PC film and CF/PC actuator; Figure S2: TGA curves of PC film, cured carbon fiber prepregs, and CF/PC actuator; Table S1: Summary of multi-stimuli responsive behavior and mechanical properties of actuator in the literature; Movies S1–S5. References [13,14,17,32,33,36,45] were cited in the supplementary materials.

## References

[1] Wang Y., Luo Z., Qian Y., Zhang W., Chen L. (2023). Monolithic MXene composites with multi-responsive actuating and energy-storage multi-functions. Chem. Eng. J..

[2] Zhang Y., Liu Y., Han D., Ma J., Wang D., Li X., Sun H. (2019). Quantum-confined-superfluidics-enabled moisture actuation based on unilaterally structured graphene oxide papers. Adv. Mater..

[3] Zuo B., Wang M., Lin B.-P., Yang H. (2019). Visible and infrared three-wavelength modulated multi-directional actuators. Nat. Commun..

[4] Charles A.D.M., Rider A.N., Brown S.A., Wang C.H. (2021). Multifunctional magneto-polymer matrix composites for electromagnetic interference suppression, sensors and actuators. Prog. Mater. Sci..

[5] Yuk H., Lin S., Ma C., Takaffoli M., Fang N.X., Zhao X. (2017). Hydraulic hydrogel actuators and robots optically and sonically camouflaged in water. Nat. Commun..

[6] Zhao Y., Xuan C., Qian X., Alsaid Y., Hua M., Jin L., He X. (2019). Soft phototactic swimmer based on self-sustained hydrogel oscillator. Sci. Robot..

[7] Hou W., Wang J., Lv J. (2023). Bioinspired liquid crystalline spinning enables scalable fabrication of high-performing fibrous artificial muscles. Adv. Mater..

[8] Roach D.J., Yuan C., Kuang X., Li V.C., Blake P., Romero M.L., Hammel I., Yu K., Qi H.J. (2019). Long liquid crystal elastomer fibers with large reversible actuation strains for smart textiles and artificial muscles. ACS Appl. Mater. Interfaces.

[9] He Y., Zhao X.Y., Rao P., Song H.M., Yang Y., Sun S.W., Zhou J.X., Chen Y.M., Tan L., Ma J.Z. (2022). Saline tolerant tough-yet-strong fiber-reinforced gel-nacre for soft actuator. Chem. Eng. J..

[10] Alipour S., Pourjavadi A., Poorghanbari M. (2023). Smart multi-stimuli responsive single- and bilayer hydrogels with combined shape memory and actuating properties for self-healable circuits and sensors. Eur. Polym. J..

[11] Ji X., Liu X., Cacucciolo V., Imboden M., Civet Y., El Haitami A., Cantin S., Perriard Y., Shea H. (2019). An autonomous untethered fast soft robotic insect driven by low-voltage dielectric elastomer actuators. Sci. Robot..

[12] Christianson C., Goldberg N.N., Deheyn D.D., Cai S., Tolley M.T. (2018). Translucent soft robots driven by frameless fluid electrode dielectric elastomer actuators. Sci. Robot..

[13] Song C., Zhang Y., Bao J., Wang Z., Zhang L., Sun J., Lan R., Yu Z., Zhu S., Yang H. (2023). Light-responsive programmable shape-memory soft actuator based on liquid crystalline polymer/polyurethane network. Adv. Funct. Mater..

[14] Xia Y., Mu T., He Y., Liu Y., Leng J. (2023). Fiber-reinforced liquid crystalline elastomer composite actuators with multi-stimulus response properties and multi-directional morphing capabilities. Compos. Part B Eng..

[15] Wu D., Zhang Y., Yang H., Wei A., Zhang Y., Mensah A., Yin R., Lv P., Feng Q., Wei Q. (2023). Scalable functionalized liquid crystal elastomer fiber soft actuators with multi-stimulus responses and photoelectric conversion. Mater. Horiz..

[16] Wang J., Liu Y., Cheng Z., Xie Z., Yin L., Wang W., Song Y., Zhang H., Wang Y., Fan Z. (2020). Highly conductive MXene film actuator based on moisture gradients. Angew. Chem. Int. Ed..

[17] Xu L., Zheng H., Xue F., Ji Q., Qiu C., Yan Q., Ding R., Zhao X., Hu Y., Peng Q. (2023). Bioinspired multi-stimulus responsive MXene-based soft actuator with self-sensing function and various biomimetic locomotion. Chem. Eng. J..

[18] He Q., Wang Z., Wang Y., Wang Z., Li C., Annapooranan R., Zeng J., Chen R., Cai S. (2021). Electrospun liquid crystal elastomer microfiber actuator. Sci. Robot..

[19] Wang Y., Huang W., Wang Y., Mu X., Ling S., Yu H., Chen W., Guo C., Watson M.C., Yu Y. (2020). Stimuli-responsive composite biopolymer actuators with selective spatial deformation behavior. Proc. Natl. Acad. Sci. USA.

[20] Das S., Singh R., Das A., Bag S., Paily R.P., Manna U. (2021). Abrasion tolerant, non-stretchable and super-water-repellent conductive & ultrasensitive pattern for identifying slow, fast, weak and strong human motions under diverse conditions. Mater. Horiz..

[21] Peng Z., Shi Y., Chen N., Li Y., Pei Q. (2020). Stable and high-strain dielectric elastomer actuators based on a carbon nanotube-polymer bilayer electrode. Adv. Funct. Mater..

[22] Yang X., Chen Y., Zhang X., Xue P., Lv P., Yang Y., Wang L., Feng W. (2022). Bioinspired light-fueled water-walking soft robots based on liquid crystal network actuators with polymerizable miniaturized gold nanorods. Nano Today.

[23] Erb R.M., Sander J.S., Grisch R., Studart A.R. (2013). Self-shaping composites with programmable bioinspired microstructures. Nat. Commun..

[24] Harrington M.J., Razghandi K., Ditsch F., Guiducci L., Rueggeberg M., Dunlop J.W., Fratzl P., Neinhuis C., Burgert I. (2011). Origami-like unfolding of hydro-actuated ice plant seed capsules. Nat. Commun..

[25] Elbaum R., Zaltzman L., Burgert I., Fratzl P. (2007). The role of wheat awns in the seed dispersal unit. Science.

[26] Li Y., Liu Q., Hess A.J., Mi S., Liu X., Chen Z., Xie Y., Smalyukh I.I. (2019). Programmable Ultralight Magnets via Orientational Arrangement of Ferromagnetic Nanoparticles within Aerogel Hosts. ACS Nano.

[27] Dong Y., Wang L., Xia N., Wang Y., Wang S., Yang Z., Jin D., Du X., Yu E., Pan C. (2021). Multi-stimuli-response programmable soft actuators with site-specific and anisotropic deformation behavior. Nano Energy.

[28] Kim H., Lee J.A., Ambulo C.P., Lee H.B., Kim S.H., Naik V.V., Haines C.S., Aliev A.E., Ovalle-Robles R., Baughman R.H. (2019). Intelligently actuating liquid crystal elastomer-carbon nanotube composites. Adv. Funct. Mater..

[29] Xu W., Dong P., Lin S., Kuang Z., Zhang Z., Wang S., Ye F., Cheng L., Wu H., Liu A. (2022). Bioinspired bilayer hydrogel-based actuator with rapidly bidirectional actuation, programmable deformation and devisable functionality. Sens. Actuators B Chem..

[30] Shian S., Bertoldi K., Clarke D.R. (2015). Dielectric elastomer based “grippers” for soft robotics. Adv. Mater..

[31] Huang Y., Jiang J., Li J., Su C., Yu Q., Wang Z., Chen N., Shao H. (2022). Light-driven bi-stable actuator with oriented polyimide fiber reinforced structure. Compos. Commun..

[32] Chen L., Zhang K., Ahn J., Wang F., Sun Y., Lee J., Cheong J.Y., Ma C., Zhao H., Duan G. (2023). Morph-genetic bamboo-reinforced hydrogel complex for bio-mimetic actuator. Chem. Eng. J..

[33] Bai L., Zhang Y., Guo S., Qu H., Yu Z., Yu H., Chen W., Tan S.C. (2023). Hygrothermic wood actuated robotic hand. Adv. Mater..

[34] Terasawa N., Asaka K. (2018). High-performance graphene oxide/vapor-grown carbon fiber composite polymer actuator. Sens. Actuators B Chem..

[35] Johannisson W., Harnden R., Zenkert D., Lindbergh G. (2020). Shape-morphing carbon fiber composite using electrochemical actuation. Proc. Natl. Acad. Sci. USA.

[36] Li S., Yang H., Zhu N., Chen G., Miao Y., Zheng J., Cong Y., Chen Y., Gao J., Jian X. (2023). Biotissue-inspired anisotropic carbon fiber composite hydrogels for logic gates, Integrated Soft Actuators, and Sensors with Ultra-High Sensitivity. Adv. Funct. Mater..

[37] Zhang X., Tian M., Raza T., Zhao H., Wang J., Du X., Zhang X., Qu L. (2021). Soft robotic reinforced by carbon fiber skeleton with large deformation and enhanced blocking forces. Compos. Part B Eng..

[38] Luo M., Tian X., Shang J., Zhu W., Li D., Qin Y. (2019). Impregnation and interlayer bonding behaviours of 3D-printed continuous carbon-fiber-reinforced poly-ether-ether-ketone composites. Compos. Part A Appl. Sci. Manuf..

[39] Mulle M., Zitoune R., Collombet F., Olivier P., Grunevald Y.-H. (2007). Thermal expansion of carbon-epoxy laminates measured with embedded FBGS – Comparison with other experimental techniques and numerical simulation. Compos. Part A Appl. Sci. Manuf..

[40] Budiman A.S., Anbazhagan S., Illya G., Song W., Sahay R., Tippabhotla S., Tay A. (2021). Enabling curvable silicon photovoltaics technology using polycarbonate-sandwiched laminate design. Sol. Energy.

[41] Liu Z., Lan X., Zeng C., Liu L., Bian W., Leng J., Liu Y. (2023). Temperature dependence analysis of mechanical properties and bending behaviors of shape memory programmable composites. Compos. Struct..

[42] Castillo F.Y., Socher R., Krause B., Headrick R., Grady B.P., Prada-Silvy R., Pötschke P. (2011). Electrical, mechanical, and glass transition behavior of polycarbonate-based nanocomposites with different multi-walled carbon nanotubes. Polymer.

[43] Luo L., Zhang F., Leng J. (2021). Multi-performance shape memory epoxy resins and their composites with narrow transition temperature range. Compos. Sci. Technol..

[44] Qi Y., Zhou C., Qiu Y., Cao X., Niu W., Wu S., Zheng Y., Ma W., Ye H., Zhang S. (2022). Biomimetic Janus photonic soft actuator with structural color self-reporting. Mater. Horiz..

[45] Yang Y., Wang H., Zhang S., Wei Y., He X., Wang J., Zhang Y., Ji Y. (2021). Vitrimer-based soft actuators with multiple responsiveness and self-healing ability triggered by multiple stimuli. Matter.

[46] Tan P., Wang H., Xiao F., Lu X., Shang W., Deng X., Song H., Xu Z., Cao J., Gan T. (2022). Solution-processable, soft, self-adhesive, and conductive polymer composites for soft electronics. Nat. Commun..

[47] Li H., Li R., Wang K., Hu Y. (2021). Dual-responsive soft actuator based on aligned carbon nanotube composite/graphene bimorph for bioinspired applications. Macromol. Mater. Eng..

[48] Qi X., Wang W., Dai H., Zhu Y., Dong Y., Fu S.-Y., Ni Q., Fu Y. (2023). Multifunctional two-way shape memory RGO/ethylene-vinyl acetate composite yarns for electro-driven actuators and high sensitivity strain sensors. Compos. Part A Appl. Sci. Manuf..

[49] Dong Y., Wang J., Guo X., Yang S., Ozen M.O., Chen P., Liu X., Du W., Xiao F., Demirci U. (2019). Multi-stimuli-responsive programmable biomimetic actuator. Nat. Commun..

[50] Chen Y., Valenzuela C., Zhang X., Yang X., Wang L., Feng W. (2023). Light-driven dandelion-inspired microfliers. Nat. Commun..

