# Supplementary material for: Ultrarobust Actuator Comprising High-Strength Carbon Fibers and Commercially Available Polycarbonate with Multi-Stimulus Responses and Programmable Deformation
Source: Polymers (Basel). 2024 Apr 19;16(8):1144. doi: 10.3390/polym16081144 (PMC11053830; doi:10.3390/polym16081144)
Supplement: Supplementary file 1 [file polymers-16-01144-s001.zip › Supporting information.pdf]

Supporting information

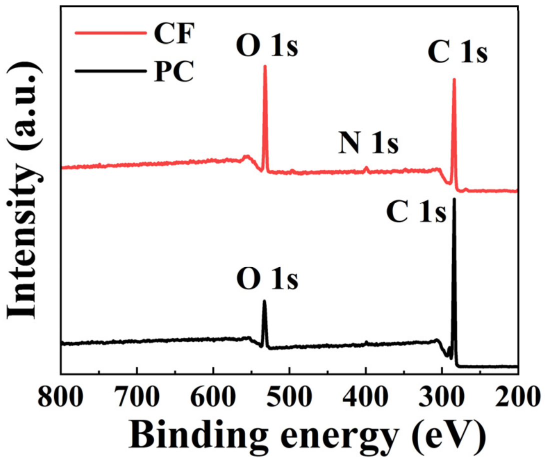

Figure S1. XPS curves of PC film and CF/PC actuator.

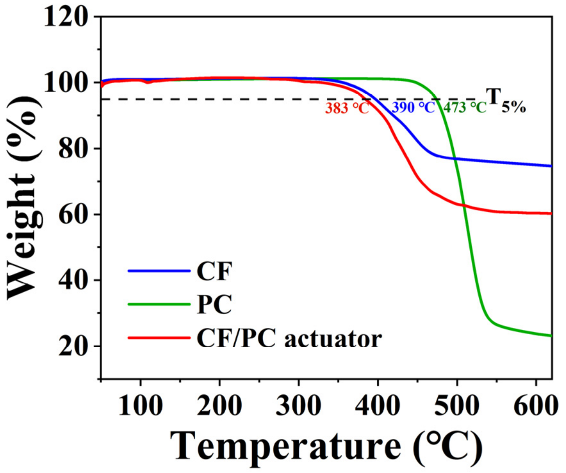

Figure S2. TGA curves of PC film, cured carbon fiber prepregs, and CF/PC actuator.

Table S1. Summary of multi-stimuli responsive behavior and mechanical properties of actuator in the literature.

Reinforcing materials	Stimulus methods	Stimulus types	Geometries		Anisotropic	Response time	Mechanical strength	Grab/ lift	Ref.
			Bend	Twist					
MXene	Heat, light, solvent	3	√	×	×	1.6 s	-	-	[17]
Bamboo fiber	Heat, solvent	2	√	√	√	4 s	5.6 MPa	203 g	[32]
Wood fiber	Heat, light, humidity	3	√	√	√	43 s	90 MPa	300 g	[33]
CFs	Heat,	2	√	√	√	-	3MPa		[36]

	electricity								
carbonized silk fabric	Heat, light, electricity, solvent	5	√	×	×	11 s	2.1 MPa	-	[45]
Liquid Crystalline	Heat, light	2	√	√	√	8 s	12 MPa		[13]
CFs	Heat, light, electricity	3	√	√	√	21 s	400 MPa	201 mg	[14]
**CFs**	**Heat, light, electricity**	**3**	√	√	√	**4 s**	**1230 MPa**	**105.8 g**	**This work**
